# Supplementary material for: Processing Conditions of a Medical Grade Poly(Methyl Methacrylate) with the Arburg Plastic Freeforming Additive Manufacturing Process
Source: Polymers (Basel). 2020 Nov 12;12(11):2677. doi: 10.3390/polym12112677 (PMC7696228; doi:10.3390/polym12112677)
Supplement: Supplementary file 1 [file polymers-12-02677-s001.pdf]

polymers

Supplementary Information

Processing Conditions of a Medical Grade Poly(Methyl Methacrylate) with the Arburg Plastic Freeforming Additive Manufacturing Process

Lukas Hentschel [1,*], Frank Kynast [2], Sandra Petersmann [3], Clemens Holzer [1] and Joamin Gonzalez-Gutierrez [1,*]

Received: 15 October 2020; Accepted: 11 November 2020; Published: date

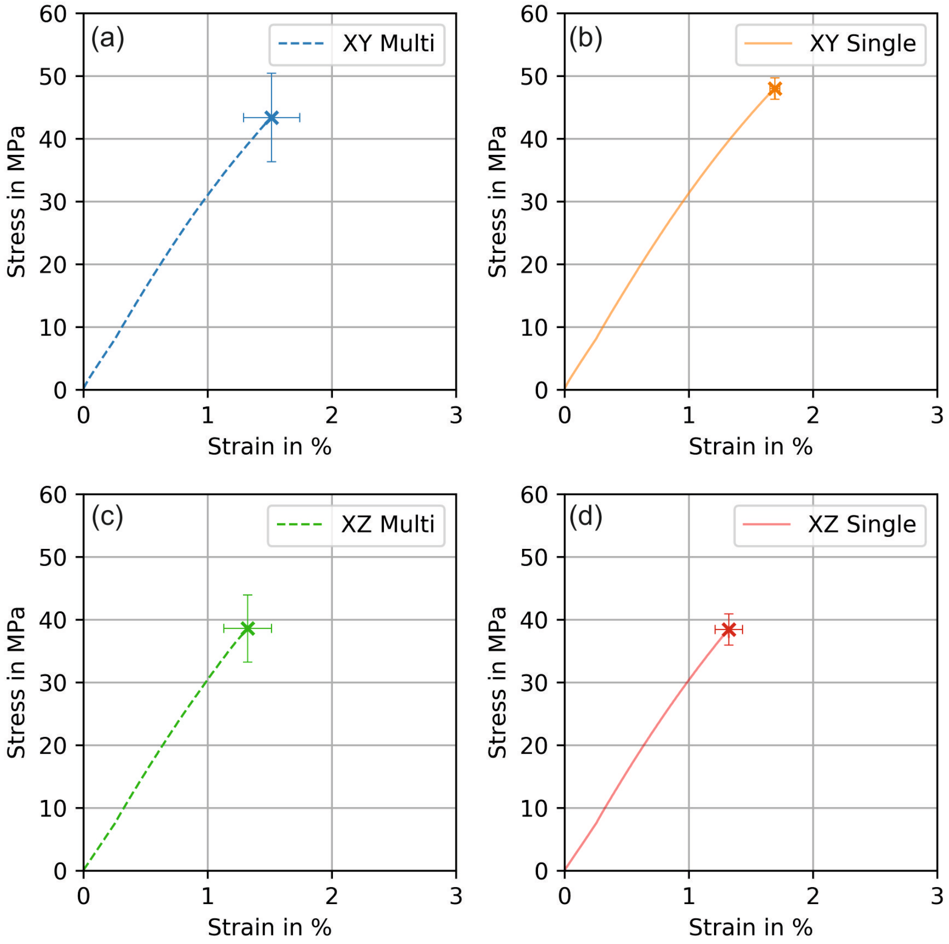

Figure S1. Stress-strain curves for the individual treatments investigated: (**a**) Print orientation XY multiple specimens per batch, (**b**) print orientation XY and single specimen per batch, (**c**) print orientation XZ and multiple specimens per batch, and (**d**) orientation XZ and single specimens per batch

Publisher's Note: MDPI stays neutral with regard to jurisdictional claims in published maps and institutional affiliations.

© 2020 by the authors. Submitted for possible open access publication under the terms and conditions of the Creative Commons Attribution (CC BY) license (http://creativecommons.org/licenses/by/4.0/).
